# Bi-objective bus scheduling optimization with passenger perception in mind

**DOI:** 10.1038/s41598-023-32997-4

**Published:** 2023-04-13

**Authors:** Shuai Liu, Lin Liu, Dongmei Pei, Jue Wang

**Affiliations:** 1 College of Geodesy and Geomatics, Shandong University of Science and Technology, Qingdao, 266590 China; 2 QingDao ZhenQing Bus Group Co., Ltd, Qingdao, China

**Keywords:** Computational science, Socioeconomic scenarios, Sustainability

## Abstract

With the development of big traffic data, bus schedules should be changed from the traditional "empirical" rough scheduling to "responsive" accurate scheduling to meet the travel needs of passengers. Based on passenger flow distribution, considering passengers' feelings of congestion and waiting time at the station, we establish a Dual-Cost Bus Scheduling Optimization Model (Dual-CBSOM) with the optimization objectives of minimizing bus operation and passenger travel costs. Improving the classical Genetic Algorithm (GA) by adaptively determining the crossover probability and mutation probability of the algorithm. We use an Adaptive Double Probability Genetic Algorithm (A_DPGA) to solve the Dual-CBSOM. Taking Qingdao city as an example for optimization, the constructed A_DPGA is compared with the classical GA and Adaptive Genetic Algorithm (AGA). By solving the arithmetic example, we get the optimal solution that can reduce the overall objective function value by 2.3%, improve the bus operation cost by 4.0%, and reduce the passenger travel cost by 6.3%. The conclusions show that the Dual_CBSOM built can better meet the passenger travel demand, improve passenger travel satisfaction, and reduce the passenger travel cost and waiting for cost. It is demonstrated that the A_DPGA built in this research has faster convergence and better optimization results.

## Introduction

The public transportation system is a vital social welfare undertaking in a city and a livelihood project that improves people's overall quality of life^1^. Public transit, as a primary form of urban transportation, significantly enhances the quality of urban life^2^. The bus scheduling plan is the essential criterion for bus operation. A scientific bus scheduling plan can effectively adjust social and economic benefits and the personal interests of passengers^3^. With the accelerated development of urbanization and the diversification of travel destinations, traditional scheduling methods based on a fixed period, a fixed departure frequency, and the experience of the workers will result in a waste of bus costs and a longer waiting time for passengers. How balance the revenue of the bus operation department and the efficiency of passenger transit has become an inevitable problem in bus schedules.

There are many studies on bus scheduling optimization at home and abroad. They have established bus scheduling optimization models based on computer vision simulation theory^4^, uncertainty theory^5^, and stochastic simulation theory^6^. The objective setting for the optimization model is divided into two categories: (1) the minimum cost of passengers^4,7^; and (2) the maximum benefit of public transport companies^6,8^. Passengers ' travel costs are divided into passengers ' waiting for time cost^9^, passengers ' traveling for time cost^10^, and passengers ' transfer waiting for time cost^7^. The operating costs of the bus company include the travel time of the buses^9^, the travel cost of the buses^5^, and the dynamic remaining capacity of the buses^11^. With the continuous advancement of technology, scholars in recent years have integrated the costs of both passengers and bus companies^5,10^. They make the goal of the model is to minimize the sum of costs for passengers and bus companies^9,12,13^. However, they all ignore passenger congestion when constructing their bus scheduling optimization models, which does not improve passenger satisfaction.

In terms of solving the scheduling optimization model, a variety of methods and algorithms have been proposed to solve the model by adjusting the departure interval, to allow the bus schedule to be reasonably adjusted to suit the passenger’s travel purpose. Traditional methods: such as periodic activity scheduling method^14^, Branch & Bound (B&B) methods^15,16^, and standard linear programming method^17^. These methods are only suitable for solving the scheduling models with small data dimensions and require high accuracy of data. When the data dimension increases, a lot of calculation costs will be incurred. At present, heuristic algorithms such as genetic algorithms are mainly used in the field to solve scheduling models. Liu et al. used the classical GA and calculated the optimal departure interval of buses to solve the bus dispatching optimization model^7^. Zuo, Niu, and others improved the classical GA and proposed an NSGA-II algorithm to solve the bus dispatching model^12,13^. Feng et al. proposed a GA with an "Elitist Preservation" strategy combining the economic method of "dynamic scoring" to solve the multi-objective scheduling model^18^. Niu and Zhang divided a day into several time blocks on average, calculated the scheduling model of each time segment separately, and designed a hybrid GA related to hierarchical crossover and mutation operations to solve the scheduling optimization model^19^.

In summary, bus scheduling optimization is currently based on objective objectives, such as minimizing the traveling cost for passengers or minimizing the operating costs for the bus company. They seldom take into account the soft objective, such as the passenger's riding feeling, and do not take this soft objective as the influence factor of establishing the public transport scheduling optimization model, so they cannot improve the passenger's riding experience. Secondly, when GA is used to solve the model in the existing research, the crossover and mutation probability of the algorithm are generally set as fixed values. The two probabilities cannot be automatically adjusted with the fitness of individuals and the dispersion of the population, which will affect the behavior and performance of the model and directly affect the convergence of the algorithm.

This research aims at the above two defects. First, soft objectives such as passenger perception are introduced based on considering the objective objectives in the public transport scheduling optimization model. The soft objectives are divided into two aspects: passenger perceptions of waiting at stops and passenger congestion perceptions within the bus. We constructed the Dual_CBSOM model by taking into account the interests of both bus operators and passengers so that the optimization results would increase the revenue of the bus company and improve passenger perception. Second, we improved the defect that the crossover and mutation probability in classical GA are fixed values, and constructed the A_DPGA algorithm. We designed a new adaptive crossover and mutation operator, and the crossover and mutation probability of the algorithm can be adjusted adaptively with the individual fitness during the optimization of the model.

## Data

### Data sources

In the process of building and solving the model, it is necessary to know some basic parameters of the target route. These parameters can be calculated by taking the bus and stop surveys, as well as through bus GPS positioning data combined with IC card swiping data. In this paper, we mainly use calculated data supplemented by observed data as the basic data to solve the model. These data are shown in Table 1.

**Table 1:** Basic data of the model.

Data classification	Data type	Advantages	Disadvantages
Observed data	Bus arrival and departure time Station and number of passengers getting on and off Waiting time of passengers Location and distribution of bus stations	Accurate, detailed, and realistic	High cost and heavy workload
Calculated data	Probability of passengers getting off Number of passengers in the bus Transfer station of passenger Time of passengers getting on	Low cost and wide coverage	Low accuracy and difficulty in obtaining

### Analysis of crowded feeling for passengers

The percentage of passengers in the bus reflects the level of overcrowding inside the bus and is the basis for classifying the level of bus service. The percentage of passengers is the number of passengers in the bus divided by the authorized number of passengers in the bus, and the level of crowding in the bus is also an important factor in whether or not passengers get on the bus at a stop. According to the reference^4^, we classify the crowded feeling of passengers in the car according to the percentage of passengers, and the results are shown in Table 2.

**Table 2:** Classification of the crowded feeling of passengers.

Grade	Percentage of passengers	Feelings of passengers
A	0–25%	Pick a seat at will
B	25–40%	Blandly pick a seat
C	40–50%	Reluctantly pick a seat
D	50–61.7%	Passengers' personal space still exists
E	61.7–75%	passengers have physical contact
F	75–100%	Passengers feel a little crowded
G	> 100%	Crowded and oppressive among passengers

## Establishment of Dual_CBSOM

### Dual_CBSOM assumptions

Model assumptions are made to capture the essence of the problem, ignore secondary factors, and achieve simplification of the actual problem, to facilitate the establishment, solution, analysis, and transplantation of the model^20^. The model assumptions not only reflect the actual situation to a certain extent but also save the computational cost of the model.

This research uses bus following survey and online search statistics to obtain the following model assumptions:

1. The buses travel at a uniform technical speed, without regard to special circumstances such as traffic accidents;
2. The travel rules of 5-day bus IC card users and mobile payment users can represent the travel rules of all bus passengers;
3. The time-consuming technical operations such as deceleration when entering the stop, acceleration when leaving the stop, and opening and closing doors are all fixed values;
4. The operating cost per unit time of the bus is not affected by factors such as the number of passengers;
5. The cost per unit time of bus passengers is the average wage per unit time of local residents.

### Parameters solving

#### Fitting function of passenger flow probability density

By processing the bus GPS positioning data, bus stop location data, and passenger IC card swiping data, we can calculate the number of people getting on the bus at different times at each stop^21^. Then combined with the bus scheduling schedule for further processing, we can get the number, time, and station of people getting on the bus in each driving direction. Combined with the proportion of the number of people who swipe the card, we can calculate the actual number of people getting on the bus at each stop in a certain driving direction. Finally, according to the calculated data, based on the period to fit the number of people getting on the bus at each stop, then get the passenger flow fitting function $f(t)$. Since there are several peak and low periods in a day for passenger travel, this paper uses multiple sine functions to fit the number of passengers, as illustrated in Formula (1).

1 $$f(t) = \sum\limits_{i = 1}^{fn} {a_{i} \sin } (b_{i} t + c_{i} )$$

For the selection of the order $fn$ of the above functions, although the larger the $fn$ is, the better the fitting effect of the scatter points are, but it will inevitably lead to the phenomenon of "over-fitting" and the increase of calculation amount. We set $fn=4$ and the function fitting effect is better. Through the fitting function of passenger flow probability density, we can calculate the number of passengers getting on the bus at any time at each station in each direction. If the fitting function of passenger flow probability density in the upward direction of a certain stop is ${f}_{e}(t)$, and the total number of people getting on the bus in the upward direction of the stop on that day is $r$, then the number of people getting on the bus from ${t}_{0}$ to ${t}_{1}$ time is ${x}_{e}$, it is calculated by Formula (2).

2 $$x_{e} = r\int_{{t_{0} }}^{{t_{1} }} {f_{e} (t)} dt$$

If ${t}_{0}$ is the time when the last bus left the stop, and ${t}_{1}$ is the time when the next bus enters the stop, the average waiting time $t\_wait$ of passengers at the stop is given by Formula (3).

3 $$t\_wait = x_{e} \times (t_{1} - t_{0} )/2$$

#### Prediction of the probability of getting off

The prediction and analysis of passenger flow and passenger flow direction for public transportation is the basis and premise of road traffic planning^22^. The parameters affecting the bus departure time are simplified to be influenced only by the time spent by passengers getting on and off the bus, i.e. the stopping time of a bus at a station is determined by the number of people getting on and off at that station. This requires the dynamic calculation of the number of people getting on and off at the station. The number of people getting on at the station can be calculated using Formula (4), and the number of people getting off at station kth is the cumulative sum of the number of people who get on the bus at previous $k-1$ stations and then get off at the kth station. Under the premise of knowing the number of people getting on the bus at the previous $k-1$ stations, the number of people getting off at the kth station can be calculated by the attraction weight of the reverse direction station and the Poisson distribution rate of passenger travel^23,24^. The Matrix $W$ of the probability of getting off the bus can be defined by the attraction weight of the reverse direction station and the passenger trip Poisson distribution, as illustrated in Formula (4).

4 $$W_{m \times m} = \left[ {\begin{array}{*{20}c} 0 & {w_{1,2} } & \cdots & {w_{1,m} } \\ 0 & 0 & \cdots & {w_{2,m} } \\ \cdots & \cdots & \ddots & \cdots \\ 0 & 0 & \cdots & 0 \\ \end{array} } \right]$$

where $m$ is the total number of stations, $W$ is an upper triangular matrix, the kth row indicates the probability of passengers getting on the bus at the kth station and getting off at the subsequent stations, and the kth column indicates the probability of passengers getting on the bus at the previous k − 1 stations and getting off at the kth station, e.g. the probability of getting on at the 2nd station and getting off at the kth station is ${W}_{2,k}$. The number of people getting off at a station can be calculated dynamically by $W$. For example, if the number of passengers getting on at stations 1st ~ k − 1 in the upward direction is $x={[x}_{1},{x}_{2},\dots ,{x}_{k-1}]$, then the number of people getting off at station k can be calculated by Formula (5):

5 $$y^{ + } = [\begin{array}{*{20}c} {x_{1} } & {\begin{array}{*{20}c} {x_{2} } & {\begin{array}{*{20}c} {...} & {x_{k - 1} } \\ \end{array} } \\ \end{array} } \\ \end{array} ] \times [\begin{array}{*{20}c} {W_{1,k} } & {\begin{array}{*{20}c} {W_{2,k} } & {\begin{array}{*{20}c} {...} & {W_{k - 1,k} } \\ \end{array} } \\ \end{array} } \\ \end{array} ]^{ - 1}$$

#### The time when the bus arrives and leaves the stop

The time $t\_jz$ of a certain bus entering the stop and the time $t\_lz$ leaving the stop are important parameters for simulating and dynamically calculating the number of people getting on and off at the stop. From model assumption (1): we know that the time $t\_j{z}_{k}$ when the bus arrives at the kth stop is the time $t\_j{z}_{k-1}$ when the bus leaves the k-1th stop plus the time it takes to travel at the technical speed from the $k-1$ stop to the $k$ stop. The time $t\_l{z}_{k}$ of the bus leaving the stop at the kth stop is the time $t\_j{z}_{k}$ that the bus arrives at the stop plus the time required for technical operations such as slowing down when arriving at the stop, accelerating when leaving the stop, opening and closing the doors, and the time it takes for passengers to get on and off the bus.

#### Bus travel speeds

The speed at which buses travel during operation depends largely on the congestion on the road. We classify and discuss the speed of buses according to the congestion in the study area. The results are shown in Table 3.

**Table 3:** Classification of bus speed.

Time	Speed (km/h)
6:00–6:30	30
6:30–8:30	20.5
8:30–17:00	30
17:00–20:00	20.2
20:00–22:00	30

In Table 3, the average bus speed is 20.5 km/h in the morning peak from 6:30 to 8:30 a.m. and 20.2 km/h in the evening peak from 17:00 to 20:00. The average bus speed is 30 km/h at other times of the day.

#### Description of other important parameters

In order to express the Dual_CBSOM more conveniently and quickly, we set i as the serial number of the bus departure and set k as the serial number of the bus stop. The settings of important parameters in the model are shown in Table 4.

**Table 4:** Description of important parameters.

Parameters	Specific forms	Description of parameters
*T*	[*t*_1_, *t*_2_, …]	Departure schedule optimized by bus schedules, where ti is the departure time of the ith bus
*S*	{*T*_1_, *T*_2_, …}	This is the population, each individual in the population is a departure timetable T
*ST*	{*ST*_1_, *ST*_2_, …}	Populations with binary encoding
*F*, *P*	[*f*_1_, *f*_2_, …], [p_1_, p_2_, …]	The fitness and selection probability of each individual in the population
*m*	28	Total number of stops on a line
*X*, *Y*	[x_1_, x_2_, …], [y_1_, y_2_, …]	The number of passengers getting on and off at the stop
*D*	[d_1_, d_2_, …]	The actual distance between adjacent stops of a line, the unit is km
*α*,*β*	*α* + *β* = 1	Weight coefficients for converting a dual objective function to a single objective function
*o*	16 s	Time-consuming for the bus to slow down when arriving at the stop, accelerate when leaving the stop, and open and close the doors
*r*_*k*_	[r_1_, r_2_, …]	The total number of passengers taking the bus in a certain direction of each stop for 1 day

### Dual_CBSOM building

For a certain line, the operating cost of the bus company and the travel cost of passengers are directly affected by the bus scheduling plan. During the same period, the more frequent the bus departures, the higher the operating cost of the bus company and the lower the waiting cost of passengers, and vice versa. The objective of this model is to find a scheduling schedule that balances the interests of both the bus company and bus passengers.

#### Establishment of the objective function

We established a bus scheduling optimization model with the first departure time of 6:00 and the last departure time of 22:00 within one day. The optimization objectives are the lowest cost of bus company and the lowest cost of passenger travel, respectively. There are the following three objective functions ${f}_{1}$, ${f}_{a2}$, and ${f}_{b2}$:

1. The operating cost of the bus company
  Knowing that a departure schedule is T and from model assumptions (3) and (4), we can calculate the operating costs of the bus company by Formula (6):
  6 $$f_{1} (T) = a_{1} \sum\limits_{i = 1}^{Len\_T} {(t_{i}^{end} } - t_{i}^{1} )$$
  In this Formula, ${a}_{1}$ is the bus operating cost per unit time, $Len\_T$ is the total number of bus departures, ${t}_{i}^{end}$ is the time when the ith bus arrives at the terminal, and ${t}_{i}^{1}$ is the departure time of the ith bus.
2. The waiting cost of passengers
  In the operational planning process of public transport, the time a passenger spends on waiting is a very critical element for judging passenger service^25^. Reference^26^ pointed out that the psychological feelings of passengers will change with the increase in waiting time. From 0 to 6 min, the passenger's state of mind is stable; from 6 to 15 min, the passenger begins to be anxious; from 15 to 35 min, the passenger's anxiety level increases, and the passenger considers giving up waiting; after 35 min, the passenger gives up waiting and changes the travel mode. To ensure that no passengers change their travel mode, this paper sets the bus departure interval to be less than 20 min, and the passenger’s waiting cost per unit time is $a$. The waiting cost for different waiting times is shown in Table 5.

**Table 5:** Passenger waiting costs.

Waiting time (min)	Waiting cost per unit time (yuan)
0–6	a
6–15	1.5*a
15–35	1.9*a
> 35	2.4*a
  The calculation method of passenger waiting cost is shown in Formula (7):
  7 $$f_{a2} (T) = a_{2} \sum\limits_{i = 1}^{Len\_T} {\sum\limits_{k = 1}^{m} {(t\_jz_{i}^{k} - t\_jz_{i - 1}^{k} )/2 \times r_{k} \times \int_{{t\_lz_{i - 1}^{k} }}^{{t\_lz_{i}^{k} }} {f_{k} (t)} dt} }$$
  In the Formula (7), ${a}_{2}$ is the unit time cost of passengers at different waiting times, and ${f}_{k}(\mathrm{t})$ is the probability density fitting function of the passenger flow at stop $k$.
3. The crowding cost of passengers
  The mood of passengers on the bus will change according to the degree of crowding in the bus, thus changing the cost of passengers per unit time to take the bus. As the level of crowding in the bus increases, passengers feel irritable and perceive time to be slower. According to reference^27^ we set the travel cost per unit time for passengers according to the percentage of passengers on the bus. The results are shown in Table 6.

**Table 6:** Passenger crowded costs.

Percentage of passengers	Unit time cost (yuan)	Crowding cost ratio (%)
0–40%	a	0
40–50%	1.08* a	7.41
50–61.7%	1.19* a	15.97
61.7–75%	1.28* a	21.88
75–100%	1.45* a	31.03
> 100%	1.57* a	36.31

The calculation method of the additional crowded cost for passengers is shown in Formula (8):

8 $$f_{b2} (T) = (a_{3} - a)\sum\limits_{i = 1}^{Len\_T} {\sum\limits_{k = 1}^{m} {(t\_lz_{i}^{k} - t\_lz_{i}^{k - 1} ) \times (r_{k} \times \int_{{t\_lz_{i}^{k - 1} }}^{{t\_lz_{i}^{k} }} {f_{k} (t)} dt} } - y)$$

In this Formula, ${a}_{3}$ is the crowded cost per unit time of passengers with different percentages for passengers on the bus, and $y$ is the number of passengers getting off the bus, $y$ is calculated according to Formula (5).

The dynamic calculation method of the time when the bus arrives at and leaves the stop is shown in Formula (9):

9 $$\left\{ \begin{gathered} t\_lz_{i}^{k} = t\_jz_{i}^{k} + \max (F[x_{i}^{k} ],F[y_{i}^{k} ]) + o/2 \hfill \\ t\_jz_{i}^{k} = t\_lz_{i}^{k - 1} + d_{k - 1} /30 \hfill \\ t\_jz_{i}^{1} = t_{i}^{1} \hfill \\ x_{i}^{k} = r_{k} \times \int_{{t\_lz_{i}^{k - 1} }}^{{t\_lz_{i}^{k} }} {f_{e} (t)} dt \hfill \\ y_{i}^{k} = \sum\limits_{j = 1}^{k - 1} {x_{i}^{j} \times W_{j,k} } \hfill \\ F[c] = 1.2 \times round(c + 1) \hfill \\ \end{gathered} \right.$$

In Formula (9), *round* indicates rounding to the nearest integer, $F$ is a defined operator, and the operator indicates the waiting time of the bus at the stop. According to the survey, the average time for each passenger to get on the bus is 1.2 s, and the bus driver needs to wait for the last passenger to get on the bus and sit or support the bus before starting the bus, that is, the stop time of the bus at the stop is the actual number of passengers to get on the bus plus one person's time. For example, F[3] means that this bus needs to stop and wait for $1.2\times (3+1)=4.8s$ at this stop before leaving for the next station.

#### Constraint conditions of the model

1. Minimum stock constraints for buses at stops
  In the actual operation of the buses, the buses will wait at the origin stop and the last stop for departure. Assuming that the origin stop is A^+^, there are ${p}_{1}$ buses, the last stop is A^-^, there are ${p}_{2}$ buses, and the driving distance between the two stops is $dist = \Sigma dj, j = 1, 2,..., m-1$. To ensure that at least one bus can be dispatched freely at both the origin stop and last stop, the departure schedule needs to satisfy the following constraint:
  10 $$\sum\limits_{i = 2}^{{p_{1} + p_{2} }} {(t_{i}^{1} } - t_{i - 1}^{1} ) \ge 2 \times \frac{dist}{v}$$
2. The constraint of adjacent departure interval
  To fully ensure the utilization efficiency of public resources and protect of the interests of bus passengers, it is mandatory to stipulate the maximum and minimum departure interval of two adjacent buses as illustrated in Formula (11):
  11 $$t\_min < t_{i}^{1} - t_{i - 1}^{1} < t\_max$$
  In summary, the bus scheduling optimization model is shown in Formula (12):
  12 $$\begin{gathered} \min \begin{array}{*{20}c} {} & {\left\{ \begin{gathered} f_{1} (T) = a_{1} \sum\limits_{i = 1}^{Len\_T} {(t_{i}^{end} } - t_{i} ) \hfill \\ f_{2} (T) = f_{a2} (T) + f_{b2} (T) \hfill \\ \end{gathered} \right.} \\ \end{array} \hfill \\ s.t\begin{array}{*{20}c} {} & { \, \left\{ \begin{gathered} \sum\limits_{i = 2}^{{p_{1} + p_{2} }} {(t_{i}^{1} } - t_{i - 1}^{1} ) \ge 2 \times \frac{dist}{v} \hfill \\ t\_min < t_{i}^{1} - t_{i - 1}^{1} < t\_max \hfill \\ \end{gathered} \right.} \\ \end{array} \hfill \\ \end{gathered}$$

## Use A_DPGA to solve the Dual_CBSOM

In the above optimization model, the objective functions are the operating cost ${f}_{1}(T)$ of the bus company and the total cost ${f}_{2}(T)$ of passenger travel, respectively. By introducing the weight coefficients $\alpha$ and $\beta$, we can obtain the overall objective function $f(T)=\alpha {f}_{1}(T)+\beta {f}_{2}(T)$ and transform the multi-objective optimization function into a single-objective optimization function. The variable to be optimized in the model is the departure timetable $T$, which is composed of discrete vectors to form the solution space, so the optimization problem is a combinatorial optimization problem. Theoretically, the combinatorial optimization problem can be solved by the B&B method developed based on the enumeration method. But in fact, this method cannot be applied to the model solution with a large data scale, and the time it takes to solve the bus scheduling problem using this method cannot be estimated. The GA is widely used in optimization problems with complex data and a large amount of computation. Using GA to solve combinatorial optimization problems can quickly search for acceptable optimal solutions and finally generate global optimal solutions.

Although the classical GA is suitable for solving complex optimization problems, it has slow convergence speed and poor local search ability, which cannot guarantee that the contemporary optimal value will be inherited by the next generation. Classical GA contains parameters such as crossover and mutation probabilities that are determined empirically and cannot be changed during the iterative process of the algorithm. AGA can improve the defects of classical GA, but AGA tends to produce a local optimal solution, and the population tends to fall into "premature maturity" in the process of solving the model and cannot find the global optimal solution. In this research, the AGA is improved and an A_DPGA is proposed to solve the Dual_CBSOM model.

### Proposal of A_DPGA

#### AGA

In the classical GA, the crossover probability and mutation probability of genes are set according to people’s experience and these two probabilities remain unchanged during the optimization process. In this paper, the AGA is used to solve the model, and the adaptive adjustment function is introduced, so that the crossover probability ${p}_{c}$ and mutation probability ${p}_{m}$ are automatically adjusted with the individual fitness and the dispersion degree of the population. When the population tends to fall into the local optimal solution, ${p}_{c}$ and ${p}_{m}$ are increased accordingly, and when the population diverges in the solution space, ${p}_{c}$ and ${p}_{m}$ are correspondingly decreased. In AGA, the solution process of ${p}_{c}$ and ${p}_{m}$ is shown in Formulas (13) and (14).

13 $$p_{c} = \left\{ \begin{gathered} k_{1} \frac{{f_{\max } - f_{1} }}{{f_{\max } - f_{avg} }},f_{1} \ge f_{avg} \hfill \\ k_{2} { , } \quad \quad \quad \quad \quad f_{1} \le f_{avg} \hfill \\ \end{gathered} \right.$$

14 $$p_{m} = \left\{ \begin{gathered} k_{3} \frac{{f_{\max } - f_{2} }}{{f_{\max } - f_{avg} }},f_{2} \ge f_{avg} \hfill \\ k_{4} { , } \quad \quad \quad \quad \quad f_{2} \le f_{avg} \hfill \\ \end{gathered} \right.$$

**Figure 1:**
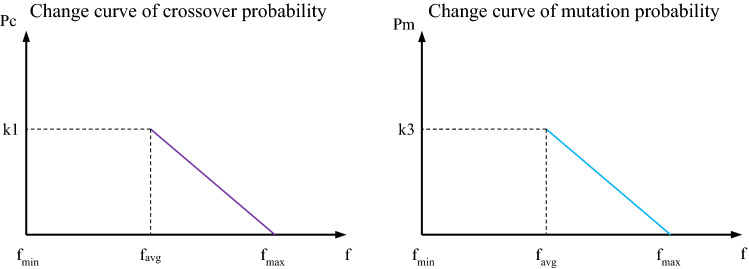
Adaptive change of crossover probability and mutation probability at AGA.

In the Formulas, ${f}_{max}$ is the largest fitness value in the group, ${f}_{avg}$ is the average fitness value in the group, ${f}_{1}$ is the larger fitness value of the two individuals to be crossed, and ${f}_{2}$ is the fitness value of the individual to be mutated. ${k}_{1}$ and ${k}_{2}$ are crossover probability constants, and ${k}_{3}$ and ${k}_{4}$ are mutation probability constants. The adaptive changes in crossover probability and mutation probability are shown in the following Fig. 1.

The two charts in Fig. 1 show the process of adaptive change of probability with the fitness value of individuals in the population in the maximum optimization problem. The left chart shows the adaptive change curve of crossover probability, and the right chart shows the adaptive change curve of mutation probability.

#### A_DPGA

Due to the shortcomings of AGA, it is easy to fall into premature convergence and destroy optimal individuals, which reduces AGA's effectiveness. In this paper, we propose an A_DPGA. The two probabilities should vary gradually at the average fitness ${f}_{avg}$ of the population, that is, the two probabilities decrease slowly with the individual's fitness changes. This increases the probability of crossover and variation of individuals at the mean fitness and reduces the likelihood of the population falling into a local optimum solution. Around the maximum fitness ${f}_{max}$ of the population, the two adjustment curves should be as smooth as they can be so that the two probabilities gradually approach zero as the individual fitness increases. This enables the better fitness individuals to be retained as much as possible in order for the results to converge more quickly to the global optimum solution. The two probabilities of IDPAGA are calculated as shown in Formula (15) and (16).

15 $$p_{c} = \left\{ \begin{gathered} \frac{{p_{c\max } - p_{c\min } }}{{1 + \exp (\frac{{2(f^{\prime} - f_{avg} )}}{{f_{\max } - f_{avg} }} - 1)}} + p_{c\min } ,f^{\prime} \ge f_{avg} \hfill \\ p_{c\max } { , } \quad \quad \quad \quad \quad f^{\prime} < f_{avg} \hfill \\ \end{gathered} \right.$$

16 $$p_{m} = \left\{ \begin{gathered} \frac{{p_{m\max } - p_{m\min } }}{{1 + \exp (\frac{{2(f - f_{avg} )}}{{f_{\max } - f_{avg} }} - 1)}} + p_{m\min } ,f \ge f_{avg} \hfill \\ p_{m\max } { , } \quad \quad \quad \quad \quad f < f_{avg} \hfill \\ \end{gathered} \right.$$

**Figure 2:**
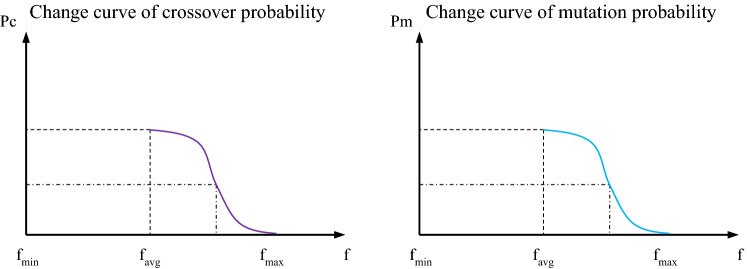
Adaptive change of crossover probability and mutation probability at A_DPGA.

Formulas (15) and (16) are the adaptive change process of crossover probability and mutation probability in the IDPAGA. The population's maximum and minimum crossover probabilities are known as ${p}_{cmax}$ and ${p}_{cmin}$, respectively. The population's maximum and minimum mutation probabilities are known as pmmax ${p}_{mmax}$ and ${p}_{mmin}$, respectively. Figure 2 displays the changes in the two probabilities.

The left and right charts in Fig. 2 are the adaptive change curves of crossover and mutation probability in the IDPAGA, respectively. At the average fitness ${f}_{avg}$ of the population, the two probabilities change slowly. At the fitness close to the maximum fitness ${f}_{max}$ of the population, the two adjustment curves become smooth.

### Solve the Dual_CBSOM

A_DPGA is an iterative calculation process. Its basic process includes initial population generation, encoding–decoding, fitness calculation, individual selection probability allocation, selection, crossover, mutation, termination condition judgment, and other processes.

Assuming that the total number of individuals in the initial population is $Sn$, the initial population can be obtained by generating $sn$ individuals. Define the departure interval in minutes. For the generation of each timetable $T$, ${t}_{1}=6:00$, ${t}_{end}=22:00$, and constraints (1) and (2) must be satisfied.

#### Encoding–decoding

We sequentially code the Individuals for the entire population. The coding principles are as follows: first, generate 960 elements coded as 0. For each ti in $T$, if the time t_i_ is x minutes away from 6:00, code the xth 0 elements as 1.

The decoding principle is as follows: in the 960 binary codes, find the position of code 1 in turn, then the position of code 1 found this time can be converted into the departure time of this time. For example, if the position of code 1 is found to be 212 bits from the starting point, and it is 212 min away from 6:00, it can be decoded to obtain $\mathrm{t}=9:32$.

#### Fitness calculation

In the GA, we can evaluate each individual in the population by the fitness function and select the individuals entering the next generation in the population by the fitness value of the individual, so the selection of the fitness function is very important. According to the DCBSOM, the optimization objective is to minimize $f(T)=\alpha {f}_{1}(T)+\beta {f}_{2}(T)$. Then define the fitness function $Fit(f(T))$:

17 $$Fit(f(T)) = 1/f(T)$$

The fitness value ${Fit}_{j}$ of each individual $j$ can be obtained by calculation.

#### Allocation and selection

The selection operation refers to the selection of individuals from the population according to a certain selection probability, and the selected "excellent" individuals with high fitness are used as parents to reproduce the next generation of individuals. The commonly used selection methods include roulette selection, random traversal sampling, local selection, truncated selection, competition selection, etc. In this paper, the roulette selection method combined with the optimal preservation strategy is used for the selection operation, and the probability of each individual being selected is as shown in Formula (18):

18 $$p_{j} = \frac{{Fit_{j} }}{{\sum\nolimits_{j = 1}^{sn} {Fit_{j} } }}$$

In the formula, ${p}_{j}$ represents the probability that the j-th individual is selected; ${Fit}_{j}$ represents the function fitness value of the jth individual; $sn$ is the population size, that is, the number of individuals in the population.

The cumulative probability for each individual is then calculated as shown in Formula (19):

19 $$Q_{j} = \sum\nolimits_{i = 1}^{j} {p_{j} }$$

In the formula, ${p}_{j}$ represents the probability that the jth individual is selected, and set ${\mathrm{p}}_{0}=0$; ${Q}_{j}$ represents the cumulative probability of the jth individual.

A random number z uniformly distributed between 0 and 1 is generated one at a time, and when ${Q}_{j-1}<z<{Q}_{j}$, the jth individual is selected as the parent. Repeat the above process, select sn-1 individuals, and then select the best individual in the population as the last individual in the parent.

#### Genetic crossover

**Figure 3:**
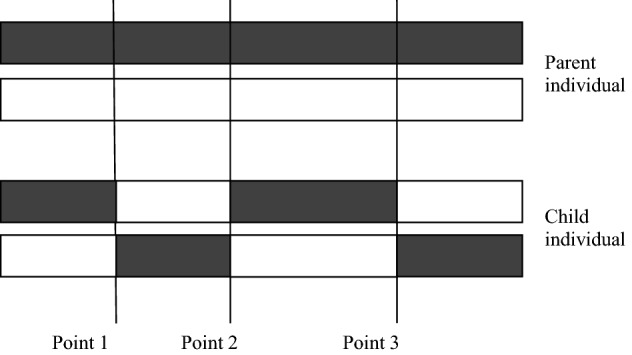
Schematic diagram of multi-point crossover.

**Figure 4:**
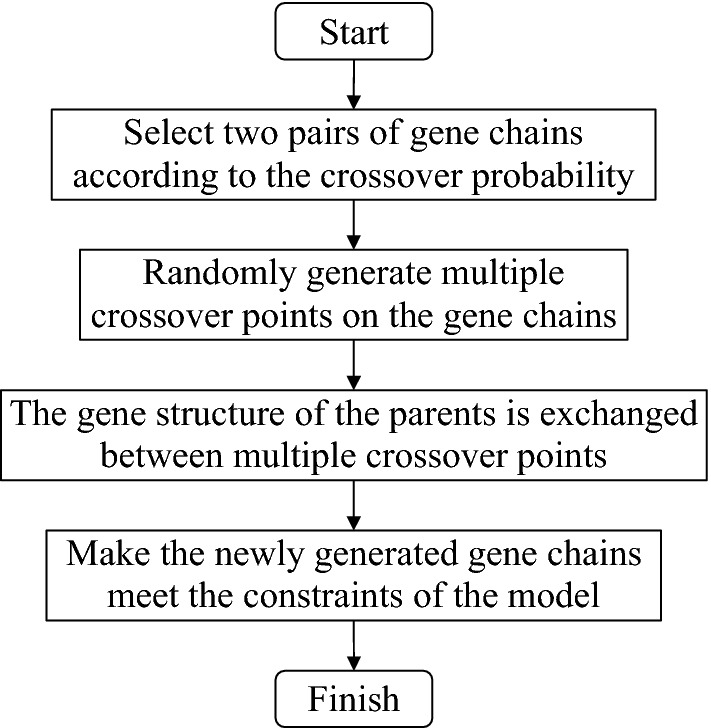
Technical route of genetic crossover.

Gene crossover is the process of crossing parts of the structures in two individuals to produce two new sub-individuals. The selection of crossover probability of the GA will affect the performance of the algorithm and directly affect the convergence of the algorithm. The higher the crossover probability, the more likely it is that the pattern of old individuals will be destroyed, and the faster new individuals will be created. However, too high a crossover probability may destroy excellent individual patterns, while too low a crossover probability may delay the generation of new individuals and lead to premature maturation of the algorithm. The common crossover methods for binary coding are single-point crossover, multi-point crossover and uniform crossover, etc. This paper uses the multi-point crossover method to solve the model. In this paper, the design of the multi-point crossover method schematic diagram is shown in Fig. 3, and the design of the crossover technique route is shown in Fig. 4.

As shown in Fig. 3, three crossover points of the genetic crossover were generated in the parent individual. The structure before point 1 and between point 2 and point 3 remains unchanged without crossover transformation. The interchange between point 1 and point 2 and between point 3 and the final structure to generate new individuals. Compared with the single-point crossover method, the multi-point crossover method has greater damage to the structure of the parent individual, allowing the algorithm to have more solutions during the genetic crossover. It can promote the search for solution space and avoid premature convergence of the algorithm.

#### Genetic mutation

**Figure 5:**
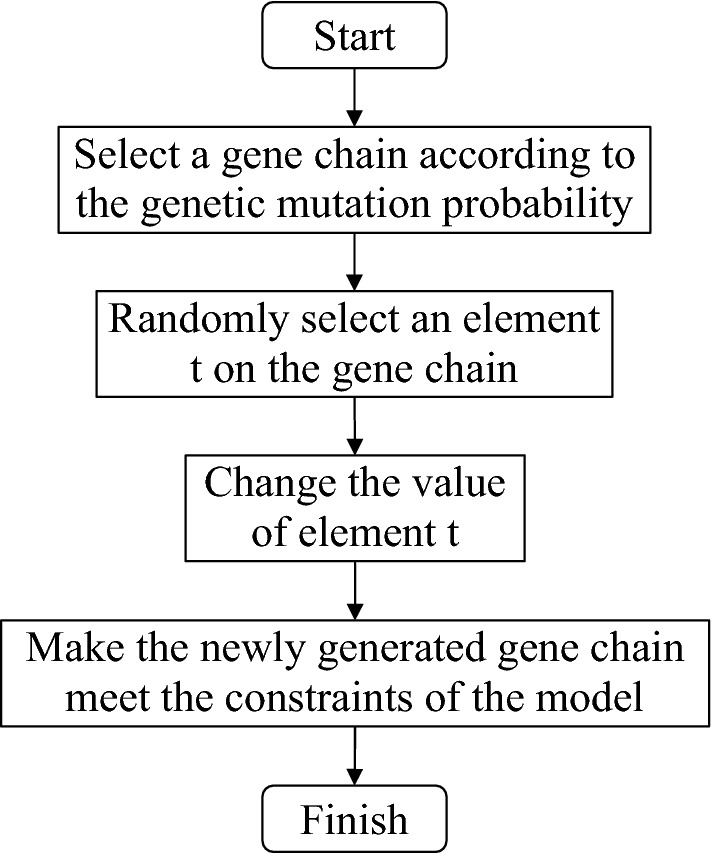
Technical route of genetic mutation.

Gene mutation is the change of the code for some individuals in the population after genetic crossover. This operation is a local search, which makes the population after genetic crossover more diverse, and can avoid the algorithm from falling into the local optimal solution. For the probability of genetic mutation, the value should not be too large, otherwise it will degrade the A_DPGA into a random search algorithm. The technical route of genetic mutation designed is shown in Fig. 5.

#### Termination condition of the algorithm

**Figure 6:**
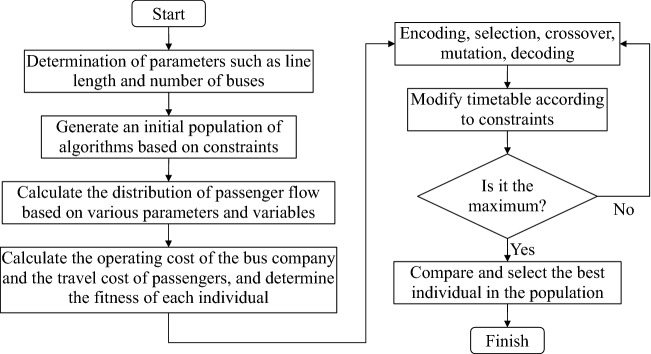
Technical route of the A_DPGA.

In general, the termination condition of a GA can be satisfied by one of the following three conditions: (1) the entire GA solution iterates a specified number of times; (2) in an iteration process, the population fitness is less than the minimum threshold we set; (3) the minimum fitness and the average fitness of the population changes for several consecutive times is less than a certain threshold. The A_DPGA used in the article sets the termination condition according to condition (1). The technical route of the A_DPGA in this paper is shown in Fig. 6.

## Example verification and analysis

**Figure 7:**
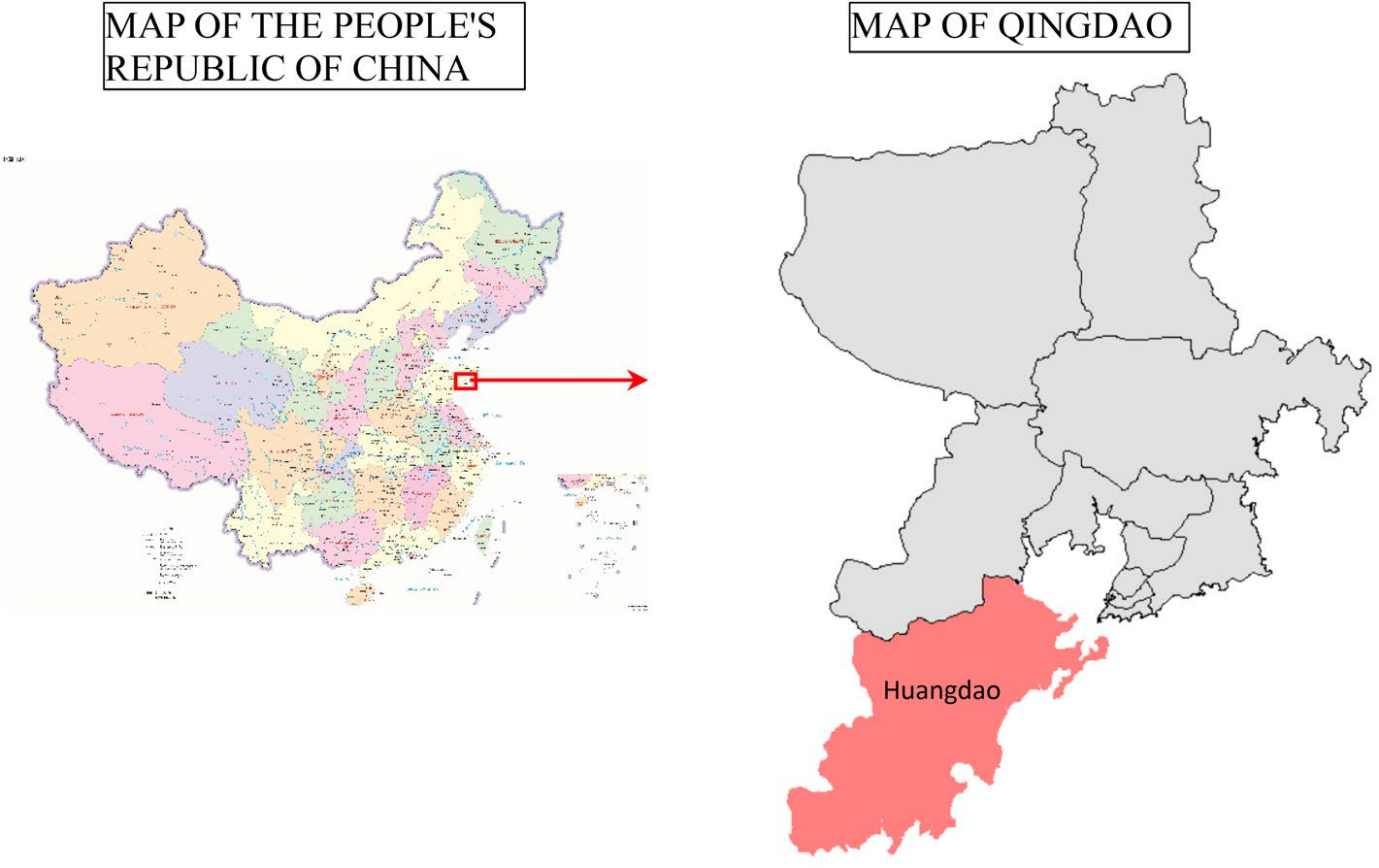
Map of the study area. Sources: the image on the left is a map downloaded from the Standard Map Service website of the National Bureau of Surverying, Mapping and Geographic Information, and the URL is http://bzdt.ch.mnr.gov.cn/index.html. The image on the right is a vectorised map generated by the first author [liu] using ArcGIS 10.7. The left map is based on the standard map with the review number of GS(2022)4316 downloaded from the standard map service website of the National Bureau of Surveying, Mapping and Geographic Information, with no modifications to the base map.

To verify the performance of the Dual_CBSOM and A_DPGA, taking the actual route in the Huangdao District of Qingdao City as an example, and studying the passenger flow data of bus schedule in a certain direction on a certain day. The map of the study area is shown in Fig. 7.

Qingdao is part of Shandong Province and is located in the southern part of the province. The red area on the left shows the location of the city for Qingdao in China, which is part of the coastal cities of eastern China. Huangdao District, also known as Qingdao West Coast New District, is located in the southwest of Qingdao and is the municipal district of Qingdao City, Shandong Province. The roads in Huangdao are generally in good condition, with high speed limits, wide lanes, and mostly tarmac roads. However, in recent years, due to the increase in the number of private cars, there are often traffic jams.

The bus line number is D9; there are 18 buses at the origin stop and the last stop, and the buses depart from these two stations. There are 28 stations in the line, and the stations passing in the upward direction are Longhu Yuanshan, Zhiyuan Middle School, …, West Coast East Bus Station, and Tangdaowan Community. The distances between stations are: [366, 704, 280, 630, 386, 530, 635, 644, 300, 778, 612, 486, 230, 479, 709, 921, 420, 718, 400, 714, 892, 320, 620, 691, 677, 355, 374], unit: M. The number of IC card swiping passengers at each station in the upward direction of the line on August 27, 2018 are [11, 529, 611, 223, 218, 210, 156, 126, 191, 174, 54, 33, 251, 158, 111, 31, 67, 27, 20, 27, 44, 13, 33, 62, 18, 7, 2, 0], unit: person-times. This data is calculated by combining the IC card swiping data and bus GPS data for 5 days from August 27, 2018, to August 31, 2018.

After investigation and actual debugging, we preset the parameters of the Dual_CBSOM as shown in Table 7:

**Table 7:** Parameter settings of the Dual_CBSOM.

Model parameter	Value	Model parameter	Value
Unit time operating cost of the bus	540 yuan/h	Crossover probability constant *k*_*1*_	0.5
Unit time cost of bus passengers	22 yuan/h	Crossover probability constant *k*_*2*_	0.9
Weight coefficient *α*	0.75	Mutation probability constant *k*_*3*_	0.01
Weight coefficient *β*	0.25	Mutation probability constant *k*_*4*_	0.05
Population size Sn	100	Maximum departure interval t_max	20 min
Maximum number of iterations Gnmax	200	Minimum departure interval t_min	5 min
The ratio of IC card-swiping passengers to total bus passengers	1:2		

**Figure 8:**
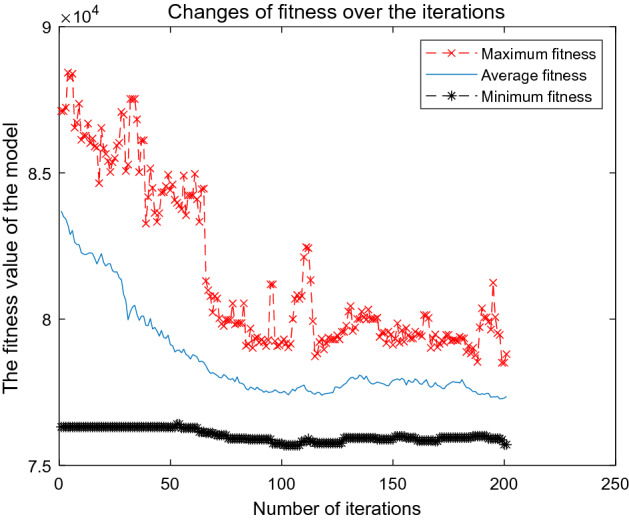
Model solving process of the GA.

**Figure 9:**
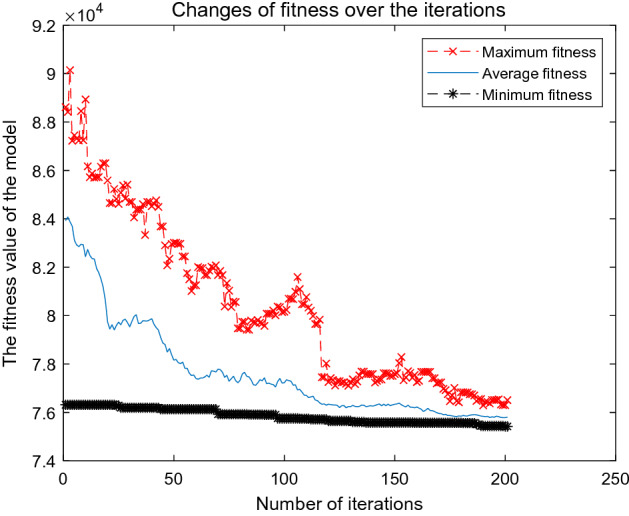
Model solving process of the AGA.

**Figure 10:**
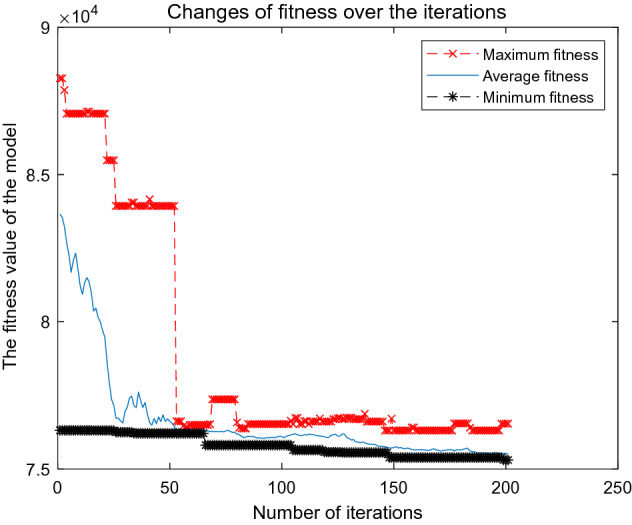
Model solving process of the A_DPGA.

**Figure 11:**
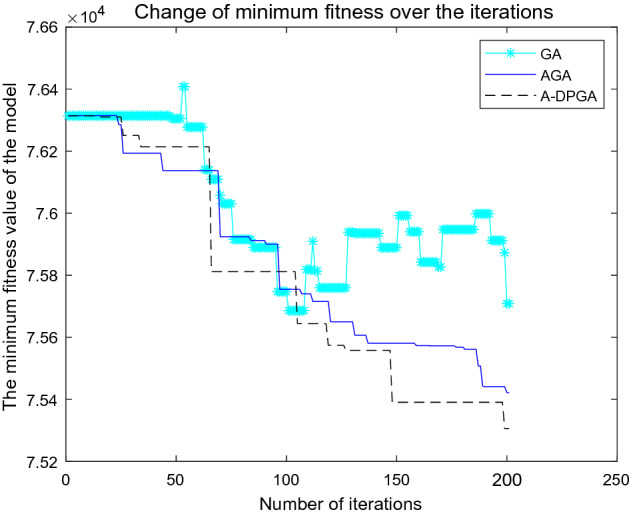
Optimal value solving process of three algorithms.

To solve the Dual_CBSOM, we each write the GA, AGA, and A_DPGA in MATLAB language. During the iteration process of the algorithm, the maximum fitness, average fitness, minimum fitness, and their corresponding individual solutions of each population are recorded. Figures 8, 9, and 10 depict the model-solving procedures for GA, AGA, and A_DPGA, respectively. Figure 11 shows the three algorithms' best solution procedures.

Figure 8 shows the change process of three fitness values with iteration times when the GA is used to solve the Dual_CBSOM. It is impossible to guarantee that the population's best individuals will be passed down to the following generation because the crossover probability and mutation probability of the population is fixed in the GA. When iterating 53 and 109 times, the population's ideal solution is destroyed, which results in a worse outcome for the current generation than for the one before it. The algorithm iterates 101 times and gets the optimal solution. The fitness of the original departure schedule is 77,085. The optimal solution of the population obtained by the GA is 75,686, which reduces 1.81%.

Figure 9 shows the changing process of fitness when solving the Dual_CBSOM with AGA. The minimum fitness of the population leveled off after 100 iterations, and the average fitness of the population oscillated and stabilized after 120 iterations. The optimal solution is 75,422 after 200 iterations, which is 2.16% less than the original departure schedule and 0.35% less than the optimal solution of GA. This shows that the AGA can better retain the excellent individuals in the population, and the fitness of the population becomes smaller and smaller with the increase of iteration times. However, the findings of this model may eventually converge to a locally optimal solution, because the method optimizes too slowly, requiring dozens of iterations to optimize a relatively minor degree of fitness value.

Figure 10 shows the solution process of the A_DPGA to the Dual_CBSOM. This figure shows that the average fitness of the population oscillates and stabilizes after 60 iterations, and the minimum fitness of the population obtains its optimal value after 199 iterations, with an optimal value of 75,306. Compared with the solution result of the original departure plan, GA and AGA, the optimal value is reduced by 2.3%, 0.5%, and 0.15%, respectively.

The three curves in Fig. 11 show the change process of the minimum fitness when the GA, AGA, and A_DPGA solve the Dual_CBSOM, respectively. The GA can not guarantee that the current optimal value can be successfully inherited by the next generation population, so the optimal value will suddenly increase in some iterations. Both the AGA and the A_DPGA can make the optimal value in the population to be inherited by the next generation population by adaptively adjusting the parameters, but the solution speed of the AGA is too slow and the time cost can be high. The A_DPGA can find the optimal value rapidly and make sure that it is more appropriate for the actual situation by smoothing the probability around the average fitness and the maximum fitness.

**Figure 12:**
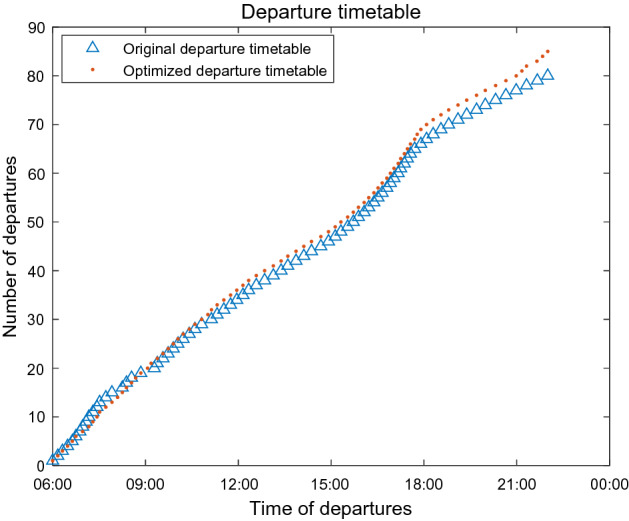
Comparison of departure timetables.

Since the optimal value obtained in this study by solving the model with the A_DPGA is better, we choose the departure timetable corresponding to this optimal value for comparison with the original departure timetable, as shown in Fig. 12.

From Fig. 12, we can see that the difference between the optimized departure timetable and the original departure timetable is mainly concentrating on three time periods: 6:30–9:00, 11:30–15:00, and 17:30–22:00. For example, the optimized departure plan increases the number of bus departures during the morning rush hour from 6:30 to 8:30 a.m. At the same time, there are other times when the number of bus departures is reduced compared with the original departure timetable. The optimized departure timetable more closely follows the passenger flow distribution law.

Table 8 shows a summary of the original departure schedule and the results solved by the three algorithms. The overall objective function value of the acceptable solution obtained by solving the model with the A_DPGA is 75,306, while the overall objective function value of the original departure timetable is 77,085, which is reduced by 2.3%. The bus operation cost *f*1 increased from 39,164 yuan to 40,798 yuan, which is a 4.0% increase in costs due to 5 more bus dispatches. Passenger cost *f*2 dropped from 190,849 yuan to 178,832 yuan, a decrease of 6.3%. The total waiting cost of passengers decreased from 96,637 yuan to 84,552 yuan, which declined by 12.51%. The waiting time per capita of passengers decreased by 1.07 min. This demonstrates that the results obtained by solving the model with the A_DPGA improve passenger perceptions, reduce passenger waiting times, reduce the occurrence of passenger crowding, and provide decision support for bus optimization.

**Table 8:** Summary of results.

	Original departure schedule	GA	AGA	A_DPGA
Total fitness value	77,085	75,686	75,422	75,306
Bus company cost (yuan)	39,164	40,849	40,100	40,798
Passenger waiting cost (yuan)	96,637	86,191	87,036	84,552
Passenger crowding cost (yuan)	94,212	94,005	94,350	94,280
Total cost of passenger travel (yuan)	190,849	180,196	181,386	178,832
Number of iterations		101	199	198
Number of departures	80	85	83	85

## Conclusion

To address the shortcomings or knowledge gaps identified in the above literature, this paper addresses bus scheduling in two main parts: First, use the original bus data to analyze and calculate the basic information of passengers, and establish a Dual_CBSOM according to the travel rules of passengers; Second, improve the classical AGA and design a new adaptive crossover operator and mutation operator, so that the crossover probability and mutation probability can automatically adjust their values according to the fitness of individuals and the degree of dispersion of the population. We use the A_DPGA to solve the Dual_CBSOM, and take the actual line data as an example to verify the feasibility of the Dual_CBSOM and A_DPGA.

The example's results show that the above method is used to optimize the bus dispatch timetable in Huangdao District, Qingdao. Compared with the original scheduling schedule, the solved scheduling schedule has higher passenger satisfaction, less waiting time, and lower extra congestion. After optimizing the bus departure schedule, passengers will be more inclined to choose the bus as their travel mode and the number of passengers will increase, which will increase both the interests of passengers and the bus company. This shows that the use of Dual_CBSOM and A_DPGA is effective for bus optimization in Qingdao and can provide a basis for bus scheduling departure plans. This can make bus schedules more intelligent, promote the construction of smart transportation and advance the development of smart cities. Therefore, it can improve the overall operational efficiency of the urban transportation system and play a very important role in the wave of smart city construction. According to the travel patterns of passengers in different regions, the models and algorithms can also be applied to the research of public transport schedules in other cities, for example, they can provide a reference for metro scheduling plans. However, the method and results cannot be applied to the scheduling of long-distance transport such as trains and airplanes.

This paper does not consider the impact of unforeseen circumstances such as road traffic accidents on bus operations. Subsequent research can consider more realistic situations, and we can incorporate the influence of traffic lights and road accidents on the time of vehicles entering and leaving the station into the scheduling model. It is also possible to quantify the unit time cost of passengers according to the different occupations of passengers, therefore the scheduling model is more suitable for the actual situation.

## Data Availability

The data that support the findings of this study are available from the first author, [Liu], upon reasonable request.

## Code availability

Code name: Data and Code 1.rar. Developer: Shuai Liu. Contact address: Shandong University of Science and Technology, 579 Qianwangang Road, Huangdao District, Qingdao City, Shandong Province. Telephone number: 15275291288. E-mail: 1135774826@qq.com. Program language: MATLAB R2018b. Source code link: https://figshare.com/articles/dataset/Data_and_Code1/22046726. https://doi.org/10.6084/m9.figshare.22046726.
